# Supplementary material for: Effectiveness and Safety of Ayurvedic Medicines in Type 2 Diabetes Mellitus Management: A Systematic Review and Meta-Analysis
Source: Front Pharmacol. 2022 Jun 8;13:821810. doi: 10.3389/fphar.2022.821810 (PMC9213670; doi:10.3389/fphar.2022.821810)
Supplement: Supplementary file 1 [file DataSheet1.zip › Supplementary Material/Forest and Funnel Plots/Zingiber officinale/LDL-C.pdf]

Zingiber officinale - LDL-C

Study	Intervention N	Intervention Mean	Intervention SD	Comparator N	Comparator Mean	Comparator SD	Mean Diff. with 95% CI	Weight (%)
Mahluji 2013	28	67.80	27.20	30	89.20	24.90	-21.40 [-34.81, -7.99]	15.53
Arablou 2014a, Arablou 2014b	33	98.90	27.20	30	112.70	37.00	-13.80 [-29.74, 2.14]	14.18
Azimi 2014, Azimi 2016	41	225.90	19.72	39	205.94	34.41	19.96 [7.75, 32.17]	16.16
Mozaffari-Khosravi 2014, Talaei 2017, Talaei 2018	40	106.10	20.78	41	102.94	19.83	3.16 [-5.68, 12.00]	17.85
Arzati 2017, Zarezadeh 2018	23	100.00	42.10	22	107.00	34.30	-7.00 [-29.50, 15.50]	10.95
Carvalho 2020	47	106.76	28.43	56	114.81	57.06	-8.05 [-25.99, 9.89]	13.14
Gholinezhad 2020	21	79.52	30.02	21	74.71	35.31	4.81 [-15.01, 24.63]	12.20
**Overall**							-2.73 [-13.74, 8.29]	

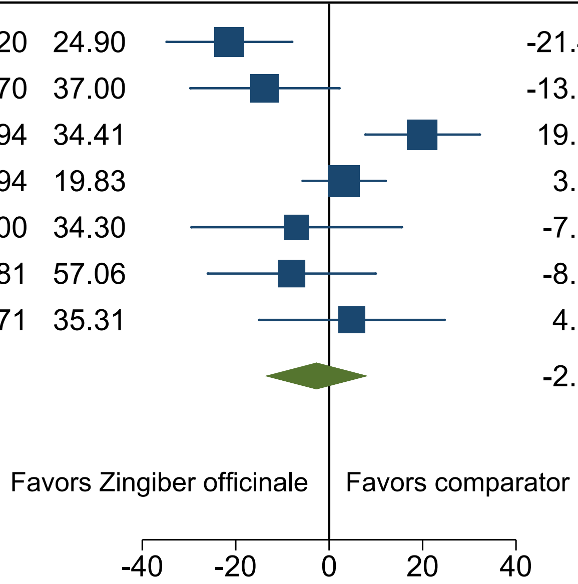

Heterogeneity: $\tau^2 = 156.65$, $I^2 = 74.49\%$, $H^2 = 3.92$

Test of $\theta_i = \theta_j$: $Q(6) = 24.72$, $p = 0.00$

Test of $\theta = 0$: $z = -0.49$, $p = 0.63$

Random-effects REML model
